# AGE DIFFERENCES IN OPEN-ENDED DECISION EVALUATIONS AND THEIR IMPLICATIONS FOR DECISION AVOIDANCE

**DOI:** 10.1093/geroni/igae098.4114

**Published:** 2024-12-31

**Authors:** Julia Nolte, Corinna Löckenhoff

**Affiliations:** Tilburg University, Tilburg, Noord-Brabant, Netherlands; Cornell University, Ithaca, New York, United States

## Abstract

Decision avoidance is more frequent with age, but associated mechanisms remain underexplored. To address this gap, a mixed-methods study captured decision makers’ subjective evaluation of choice options prior to making or avoiding choices. An adult lifespan sample (N = 227, 18-88 years, M = 50.71, SD = 19.22, 55% women) evaluated four health and consumer decision scenarios. Specifically, participants were asked to write down what they liked and disliked about the available choice options before making or avoiding a choice. Decision avoidance was positively associated with age. Open-ended responses were on average 33.08 words long (SD = 22.38) and contained a sum of 21.18 statements (SD = 16.42). The number of words or statements was unrelated to age. Responses were coded for remarks about positive (κ =.79-.88), negative (κ =.60-.74), and fixable/acceptable (κ =.32-.85) choice attributes, as well as choice preferences (κ =.34-.67). No significant findings emerged with respect to the latter two categories; among the former, the number of negative remarks dominated the positive ones. Decision makers who focused relatively more on negative attributes were older and reported poorer mental well-being. Among middle-aged and older adults, but not younger adults, a stronger focus on negative compared to positive attributes was associated with more pronounced decision avoidance tendencies. All findings remained significant after accounting for the number of words or statements. In combination, this may suggest that older age groups hold stricter decision standards or are less willing to trade-off between favorable and unfavorable choice attributes.

